# R2R3-MYB Transcription Factor SmMYB52 Positively Regulates Biosynthesis of Salvianolic Acid B and Inhibits Root Growth in *Salvia miltiorrhiza*

**DOI:** 10.3390/ijms22179538

**Published:** 2021-09-02

**Authors:** Rao Yang, Shengsong Wang, Haolan Zou, Lin Li, Yonghui Li, Donghao Wang, Hongxing Xu, Xiaoyan Cao

**Affiliations:** 1 Key Laboratory of the Ministry of Education for Medicinal Resources and Natural Pharmaceutical Chemistry, National Engineering Laboratory for Resource Development of Endangered Crude Drugs in Northwest of China, Shaanxi Normal University, Xi’an 710062, China; yangrao@snnu.edu.cn (R.Y.); shengsongwang@snnu.edu.cn (S.W.); zhl2019@snnu.edu.cn (H.Z.); shidalilin@snnu.edu.cn (L.L.); wangdonghao@snnu.edu.cn (D.W.); 2 College of Life Science, Luoyang Normal University, Luoyang 471934, China; huiyongli8209@126.com

**Keywords:** auxin, hormone, jasmonate acid, root growth, salvianolic acid B, *Salvia miltiorrhiza*, *Sm*MYB52

## Abstract

The dried root of *Salvia miltiorrhiza* is a renowned traditional Chinese medicine that was used for over 1000 years in China. Salvianolic acid B (SalB) is the main natural bioactive product of *S. miltiorrhiza*. Although many publications described the regulation mechanism of SalB biosynthesis, few reports simultaneously focused on *S. miltiorrhiza* root development. For this study, an R2R3-MYB transcription factor gene (*SmMYB52*) was overexpressed and silenced, respectively, in *S. miltiorrhiza* sterile seedlings. We found that *SmMYB52* significantly inhibited root growth and indole-3-acetic acid (IAA) accumulation, whereas it activated phenolic acid biosynthesis and the jasmonate acid (JA) signaling pathway. Quantitative real-time polymerase chain reaction (qRT-PCR) analyses revealed that *Sm*MYB52 suppressed the transcription levels of key enzyme-encoding genes involved in the IAA biosynthetic pathway and activated key enzyme-encoding genes involved in the JA and phenolic acid biosynthesis pathways. In addition, yeast one-hybrid (Y1H) and dual-luciferase assay showed that *Sm*MYB52 directly binds to and activates the promoters of several key enzyme genes for SalB biosynthesis, including *SmTAT1*, *Sm4CL9*, *SmC4H1*, and *SmHPPR1*, to promote the accumulation of SalB. This is the first report of a regulator that simultaneously affects root growth and the production of phenolic acids in *S. miltiorrhiza*.

## 1. Introduction

The rhizome of *Salvia miltiorrhiza*, referred to as Danshen in Chinese traditional medicine, contains hydrophilic phenolic acids and lipophilic tanshinones, which are major bioactive constituents that were well-studied over the last few years [1]. Phenolic acids in *S. miltiorrhiza* primarily include two important naturally bioactive products, rosmarinic acid (RA) and salvianolic acid B (SalB), which play important roles in improving microcirculation and inhibiting platelet aggregation [2,3]. Due to their remarkable pharmacological activities and growing market demand, the regulation of SalB biosynthesis in *S. miltiorrhiza* recently garnered significant attention [4,5]. SalB biosynthesis involves the phenylpropanoid- and tyrosine-derived pathways [6]. Except for the enzymes engaged in the SalB biosynthetic pathways, the upstream transcription factors (TFs) also have a great impact on the biosynthesis of SalB [7].

MYB (myeloblastosis) TFs with various functions are widely distributed in plants [8], which can be divided into four subfamilies (1R-MYB, R2R3-MYB, 3R-MYB, and 4R-MYB) according to the number and location of MYB repeats [9]. The R2R3-MYB family functions in a variety of plant-specific regulatory networks that control the development, secondary metabolism, stress mechanisms, and responses to biotic and abiotic stresses [8,9,10,11]. In *Arabidopsis thaliana*, the R2R3-MYB TF gene *AtMYB93* can be induced by auxin, and it negatively regulates lateral root development by interacting with lateral-root-promoting ARABIDILLO proteins [12].

In purple carrots, a cluster of R2R3-MYB TFs identified by quantitative trait locus (QTL) affects anthocyanin biosynthesis and modification in the root and petiole [13]. MdMYB28 decreases flavonoid accumulation in apples [14], whereas in pears, overexpressed *PpMYB17* increases flavonoid biosynthesis by activating the enzyme-encoding genes [15]. In response to brown planthopper feeding, *OsMYB30* is induced in rice and then upregulates the expression of phenylalanine ammonialyase genes, leading to increased biosynthesis and accumulation of salicylic acid and lignin [16]. The ectopic expression of *ThMYB8*, which is a R2R3-MYB TF from *Tamarix hispida*, significantly increases root growth, fresh weight, and seed germination rate compared with that of the wild-type under salt stress [17].

In *S. miltiorrhiza*, a total of 110 R2R3-MYBs were identified and classified into 37 subgroups which serve as critical regulators in the biosynthesis of secondary metabolites [18]. The overexpression of *SmMYB1* in *S. miltiorrhiza* hair root promotes phenolic acid accumulation and activates the expression of genes that encode key enzymes in the phenolic acid and anthocyanin biosynthesis pathways [19]. Similarly, SmMYB2 binds to the promoter of the salvianolic acid biosynthetic gene *CYP98A14* and increases the RA and SalB contents [20].

The methyl jasmonate (MeJA)-responsive gene *SmMYB97* promotes phenolic acid and tanshinone biosynthesis and is repressed by the JASMONATE ZIM-domain (JAZ) protein *Sm*JAZ8 [21]. Another R2R3-MYB member, *Sm*MYB111, can form a potential ternary transcription complex (*Sm*TTG1-*Sm*MYB111-*Sm*bHLH51) and positively regulates the production of SalB in *S. miltiorrhiza* [22]. The overexpression of *SmMYB36* promotes the accumulation of tanshinone, whereas it inhibits the biosynthesis of phenolic acid and flavonoid in the hairy roots of *S. miltiorrhiza* [23].

The activation of secondary plant metabolism often promotes defense and initiates growth inhibition and biomass reduction, which are referred to as tradeoffs between growth and defense. Finding the appropriate balance between root development and secondary metabolic accumulation is critical for the cultivation and breeding of *S. miltiorrhiza*. Most recently, researchers have begun to focus simultaneously on the accumulation of active compounds and the root growth. In laccase genes-knockout lines, the growth of hairy roots was inhibited and the accumulation of RA and SAB was decreased in *S. miltiorrhiza* [24]. The combination of blue and red LED light was observed to improve the growth of *S. miltiorrhiza* and promoted the accumulation of phenolic acid [25]. Nevertheless, no reports exist to date that investigate how R2R3-MYB simultaneously regulates root growth and the synthesis of secondary metabolites in *S. miltiorrhiza*.

Here, we identified *Sm*MYB52 as a regulator of root growth and SalB accumulation in *S. miltiorrhiza* through gain- and loss-of-function analysis. We found that *Sm*MYB52 positively regulated the biosynthesis of SalB by directly binding to and activating key enzyme genes in the phenolic acid biosynthesis pathway. Meanwhile, the root growth was inhibited with higher *Sm*MYB52 transcript levels, which were possibly due to the effects of *Sm*MYB52 on the biosynthesis of indole-3-acetic acid (IAA) and jasmonate acid (JA). To the best of our knowledge, this is the first report to describe the involvement of R2R3-MYB TF in the regulation of root growth and secondary metabolite accumulation in *S. miltiorrhiza*. Our results suggested that *Sm*MYB52 is a potential regulator of auxin-JA crosstalk in root development.

## 2. Results

### 2.1. SmMYB52 Impedes S. miltiorrhiza Root Growth

**Figure 1:**
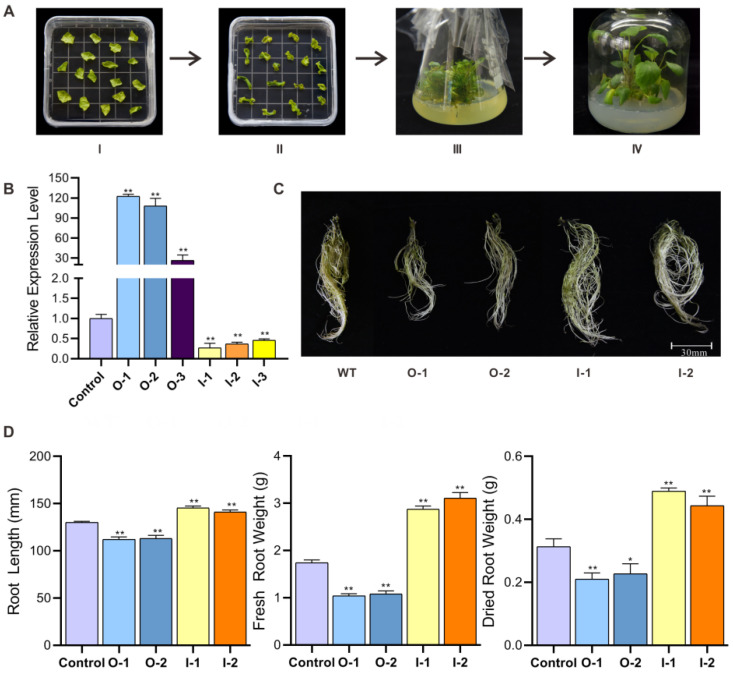
Acquisition and root phenotypes of transgenic lines. (**A**) Generation of transgenic plantlets of *S. miltiorrhiza*. (I) Preculture of *S. miltiorrhiza* leaves. (II) Callus formation on selection medium following *Agrobacterium*-mediated transformation. (III) Adventitious buds formed on selection medium. (IV) Generation of transgenic plantlets on 1/2 MS medium. (**B**) Transcript level of SmMYB52 in transgenic and control lines. O-1, O-2, and O-3 are SmMYB52 overexpression lines; I-1, I-2, and I-3 are SmMYB52 silencing lines. (**C**) Root phenotypes of transgenic and control lines were cultured on 1/2 MS medium for two months. (**D**) Root length, fresh root weight, and dried root weight of transgenic and control seedlings. All data are means of three biological replicates, and error bar represents SD; statistical significance was evaluated with Student’s *t*-test (** *p* < 0.01; * *p* < 0.05).

*SmMYB52*, which encodes an R2R3-MYB TF that is closely related to Arabidopsis AtMYB93, showed the highest transcript level in the root [26]. To further explore the function of *SmMYB52*, *SmMYB52*-overexpression and *SmMYB52*-silencing transgenic *S. miltiorrhiza* plantlets were generated via *Agrobacterium*-mediated transformation (Figure 1A and Figure S1). The expression levels of *SmMYB52* in the selected lines were evaluated by qRT-PCR (Figure 1B). We found that the transgenic lines exhibited obvious differences in root phenotype (Figure 1C). Compared with that of the control plants, the root length, fresh root weight, and dried root weight were significantly decreased in *SmMYB52*-overexpressing lines (O-1 and O-2). In contrast, *SmMYB52*-silencing lines (I-1 and I-2) exhibited a significant increase in these phenotypes (Figure 1D). These results indicated that *SmMYB52* inhibited root growth in *S. miltiorrhiza*.

### 2.2. SmMYB52 Suppresses the Biosynthesis of IAA in S. miltiorrhiza

**Figure 2:**
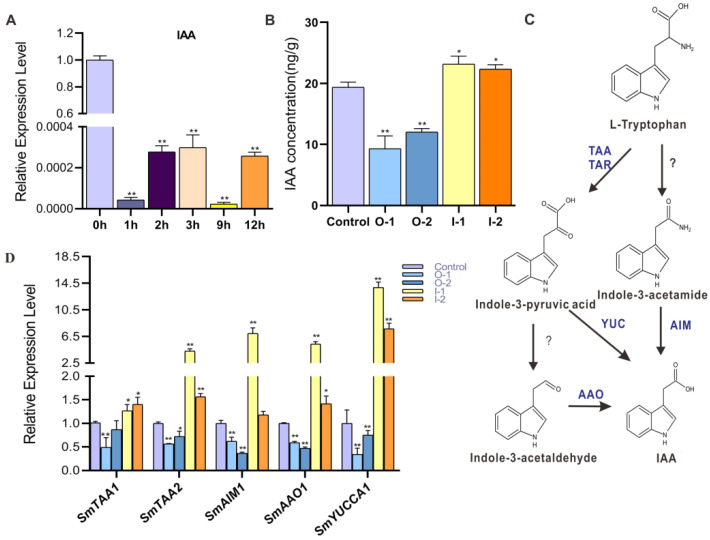
Effects of overexpressing and silencing *SmMYB52* on IAA biosynthesis (**A**) Transcript level of *SmMYB52* in response to exogenous IAA treatment. (**B**) Concentration of endogenous IAA in fresh roots of transgenic and control lines. (**C**) Proposed IAA biosynthetic pathway (trp-dependent pathway). TAA, Tryptophan aminotransferase of Arabidopsis; TAR, Tryptophan aminotransferase related; YUC, YUCCA; AIM1, Indole-3-acetamide hydrolase; AAO1, Aldehyde oxidase. (**D**) Transcript levels of key enzyme-encoding genes in IAA biosynthesis pathway. *SmTAA1* (SMil_00008622-RA_Salv), *SmTAA2* (SMil_00008177-RA_Salv), *SmYUC1* (SMil_00002467-RA_Salv), *SmAIM1* (SMil_00009945-RA_Salv), and *SmAAO1* (SMil_00014441-RA_Salv) were retrieved from the web portal at http://www.ndctcm.org/shujukujieshao/2015-04-23/27.html (accessed on 7 January 2021). All data are means of three biological replicates, and the error bar represents SD; statistical significance was evaluated with Student’s *t*-test ** *p* < 0.01; * *p* < 0.05).

Since Arabidopsis *AtMYB93* is induced by auxin, which is involved in the regulation of root growth [12,27], and is required for normal auxin responses, we hypothesized that *SmMYB52* (the homeotic gene of *AtMYB93*) may respond to auxin treatment and be associated with the biosynthesis of auxin in *S. miltiorrhiza*. Interestingly, the expression levels of *SmMYB52* were significantly suppressed by exogenous IAA treatment at all time-points from 0.5 h to 12 h (Figure 2A). The concentrations of endogenous IAA in the fresh roots of transgenic plantlets and the control were evaluated. The results revealed that compared with that of the control, the IAA levels decreased significantly in the O-1 and O-2 lines but increased considerably in the I-1 and I-2 lines (Figure 2B).

The transcript levels of key enzyme-encoding genes for the IAA biosynthesis pathway were analyzed by qRT-PCR, with the results showing that the expression levels of *SmTAA2*, *SmAIM1*, *SmAAO1*, and *SmYUC1* genes were significantly downregulated in O-1 and O-2 lines. In contrast, the expression levels of *SmTAA1*, *SmTAA2*, and *SmYUC1* were significantly upregulated in the SmMYB52-silencing lines (Figure 2C,D). Furthermore, we found that the promoter regions of these genes contained MYB-binding sites (Figure S2A).

### 2.3. SmMYB52 Affects the Biosynthesis of JA in S. miltiorrhiza

**Figure 3:**
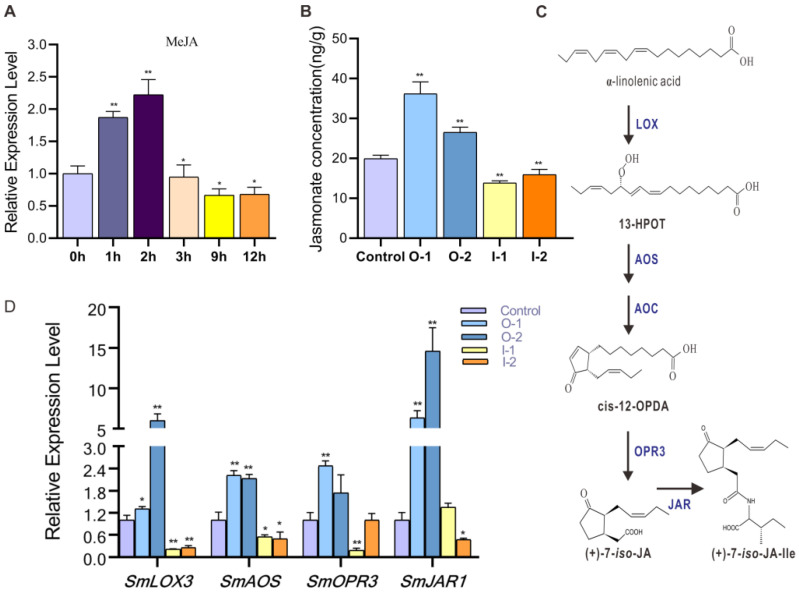
Effects of overexpressing and silencing *SmMYB52* on JA biosynthesis (**A**) Transcript level of *SmMYB52* in response to exogenous MeJA treatment. (**B**) Concentration of endogenous JA in fresh roots of transgenic and control lines. (**C**) Proposed biosynthetic pathway for JA. LOX, Lipoxygenase; AOS, Allene oxide synthase; AOC, Allene oxide cyclase; OPR3, 12-oxophytodienoate reductase 3; JAR1, JA-amino acid synthase. (**D**) Transcript levels of key enzyme-encoding genes in JA biosynthesis pathway. *SmLOX* (SMil_00027821-RA_Salv), *SmAOS* (SMil_00002529-RA_Salv), *SmOPR3* (KF220568.1), and *SmJAR1* (SMil_00003673-RA_Salv) were retrieved from the web portal at http://www.ndctcm.org/shujukujieshao/2015-04-23/27.html (accessed on 7 January 2021). All data are means of three biological replicates, and the error bar represents SD; statistical significance was evaluated with Student’s *t*-test (** *p* < 0.01; * *p* < 0.05).

A previous study reported that JA had a negative effect on adventitious root formation and root development [28]. To explore the relationship between JA and *SmMYB52*, we analyzed the promoter region of *SmMYB52* and found that the CGTAC- and TGACG-motifs, which are MeJA-responsiveness elements, were present (Figure S3). Meanwhile, the exogenous MeJA treatment significantly activated the expression of *SmMYB52* in the first 3 h (Figure 3A). The endogenous JA contents of the transgenic lines were also analyzed, and the results indicated that the JA levels in the O-1 and O-2 lines were significantly higher than that of the control. Conversely, those in the I-1 and I-2 lines were significantly lower than in that of the control (Figure 3B).

*SmJAR1*, *SmAOS*, *SmLOX3*, and *SmOPR3* are key enzyme-encoding genes involved in the jasmonic acid biosynthesis pathway in *S. miltiorrhiza* [29]. The qRT-PCR results showed that the expression levels of *SmLOX3*, *SmAOS*, and *SmJAR1* were significantly upregulated in the *SmMYB52*-overexpression lines. In contrast, *SmLOX3* and *SmAOS* were significantly downregulated in the *SmMYB52*-silencing lines (Figure 3C,D).

### 2.4. SmMYB52 Positively Regulates the Biosynthesis of SalB in S. miltiorrhiza

**Figure 4:**
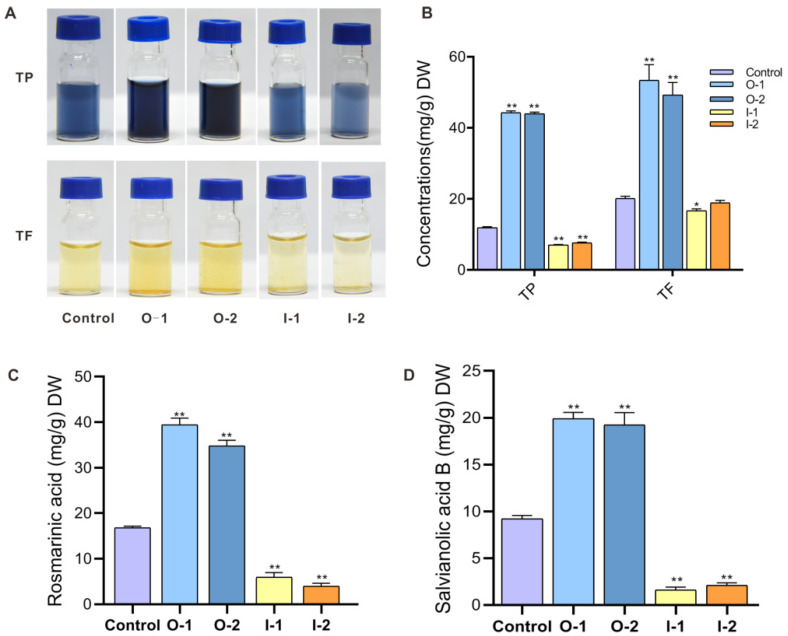
Determination of total phenolic (TP), total flavonoid (TF), rosmarinic acid, and salvianolic acid B concentration. (**A**) Color of root extracts for detection of TP and TF. (**B**) Concentration of TP and TF in dried roots of two-month-old transgenic and control lines. (**C**,**D**) Concentrations of rosmarinic acid and salvianolic acid B, respectively, in the dried roots of two-month-old transgenic and control lines. All data are means of three biological replicates, and error bar represents SD; statistical significance was evaluated with Student’s *t*-test (** *p* < 0.01; * *p* < 0.05).

The application of exogenous MeJA can promote the accumulation of RA and SalB in the hairy roots of *S. miltiorrhiza* [30]. We speculated that *SmMYB52* might regulate the production of secondary metabolites by activating JA biosynthesis. To functionally verify the effects of *SmMYB52* on the accumulation of secondary metabolites, we measured the levels of total flavonoid (TF) and total phenolic (TP) in the roots of transgenic plantlets and the control. The results showed that the levels of TP and TF in the *SmMYB52*-overexpression lines were significantly higher than that of the control, but only slightly decreased in the *SmMYB52*-silencing lines (Figure 4A,B). Therefore, the overexpression of *SmMYB52* promoted the accumulation of TP and TF in *S. miltiorrhiza*.

Subsequently, we determined the contents of RA and SalB in the roots of the transgenic lines by high-performance liquid chromatography (HPLC). Compared to that of the control, the content of RA in the O-1 and O-2 lines increased 2.3- and 2.1-fold, respectively, while it decreased to 35.3% and 23.9% in I-1 and I-2 lines, respectively (Figure 4C). Similarly, the SalB concentrations increased significantly (2.1–2.2-fold) and decreased (4.5–5.5-fold) in the *SmMYB52*-overexpression and *SmMYB52*-silencing lines, respectively (Figure 4D). These results implied that *SmMYB52* positively regulated the accumulation of RA and SalB.

**Figure 5:**
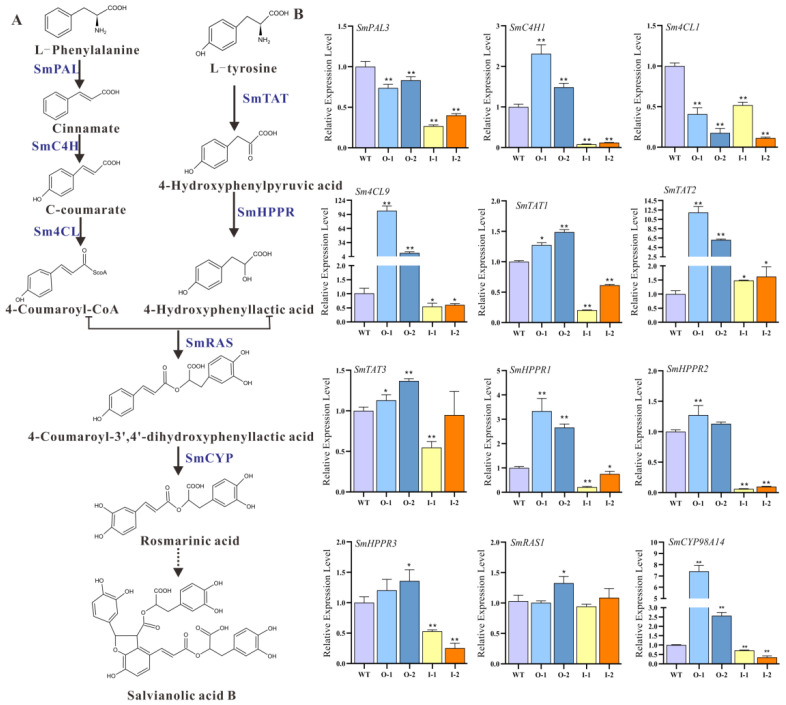
Transcript levels of enzyme-encoding genes involved in phenolic acid biosynthesis pathway in transgenic and control lines. (**A**) Proposed pathway for salvianolic acid (SalB) biosynthesis. PAL, Phenylalanine ammonia lyase; C4H, Cinnamate 4-hydroxylase; 4CL, 4-coumarate-CoA ligase; TAT, Tyrosine aminotransferase; HPPR, 4-hydroxyphenylpyruvate reductase; RAS, Rosmarinic acid synthase; CYP, Cytochrome P450-dependent monooxygenase. (**B**) Transcript levels of key enzyme-encoding genes involved in SalB biosynthetic pathway in roots of two-month-old transgenic and control lines. All data are means of three biological replicates, and the error bar represents SD; statistical significance was evaluated with Student’s *t*-test (** *p* < 0.01; * *p* < 0.05).

To further investigate the possibility that *SmMYB52* regulates phenolic acid biosynthesis, we analyzed the transcript levels of 12 phenolic acid biosynthetic genes using qRT-PCR (Figure 5A,B). Among the 12 genes, *SmC4H1*, *Sm4CL9*, *SmTAT1*, *SmTAT3*, *SmHPPR1*, *SmHPPR2*, *SmHPPR3*, and *SmCYP98A14* were significantly upregulated in the *SmMYB52*-overexpression lines and significantly reduced in the *SmMYB52*-silencing lines. Overall, these results indicated that *SmMYB52* promoted the accumulation of SalB by activating its biosynthetic pathway.

### 2.5. SmMYB52 Binds Directly to the Promoters of SmTAT1, SmHPPR1, SmC4H1, and Sm4CL9, and Activates their Transcription

**Figure 6:**
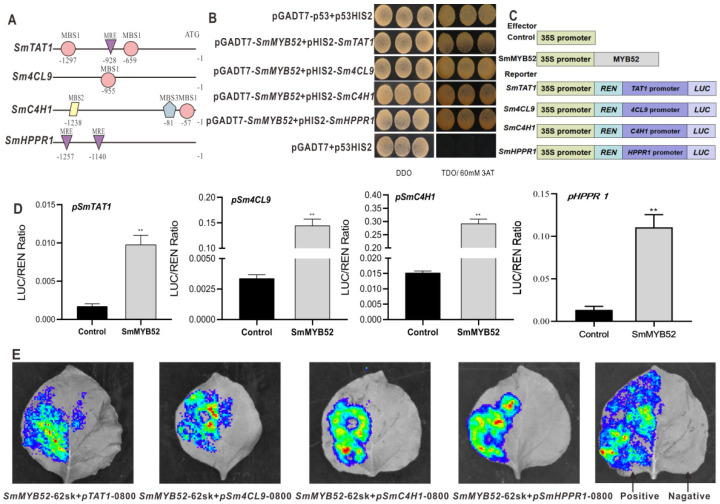
SmMYB2 binds to and activates the promoters of *SmTAT1*, *Sm4CL9*, *SmC4H1*, and *SmHPPR1*. (**A**) MYB-binding sites in promoter regions of *SmTAT1*, *Sm4CL9*, *SmC4H1*, and *SmHPPR1*. (**B**) Yeast one-hybrid assay results indicated interactions between SmMYB52 and promoter regions of *SmTAT1*, *Sm4CL9*, *SmC4H1*, and *SmHPPR1*. (**C**) Schematic diagram of constructs used in dual-luciferase assay. (**D**) Luciferase activity (LUC) analysis implied that SmMYB52 activated transcription of *SmTAT1*, *Sm4CL9*, *SmC4H1*, and *SmHPPR1*. Relative LUC activities were normalized to the renilla luciferase (REN) activity. (**E**) Transient expression assay in tobacco leaf; leaf images were photographed at 48 h following infiltration. SmbHLH37+*pSmTAT1* and SmMYB52+pGreenII 0800 LUC served as positive and negative controls, respectively. All data are means of three biological replicates, and the error bar represents SD; statistical significance was evaluated with Student’s *t*-test (** *p* < 0.01).

MYB TFs typically bind to the cis-elements of the promoters and activate or repress the expression of the enzyme genes to regulate the biosynthesis of secondary metabolites [23]. To explore the potential mechanism of how SmMYB52 regulates the biosynthesis of SalB, we analyzed the promoter sequences of enzyme genes for the phenolic acid biosynthesis pathway and found MYB-binding elements in *SmTAT1*, *Sm4CL9*, *SmC4H1*, and *SmHPPR1* (Figure 6A). Y1H assay results indicated that SmMYB52 binds directly to the promoters of *SmTAT1*, *Sm4CL9*, *SmC4H1*, and *SmHPPR1* (Figure 6B).

The promoter regions of these genes were then fused with the luciferase gene (*LUC*) to serve as reporters, whereas SmMYB52 functioned as an effector (Figure 6C). A dual-luciferase transient expression assay showed that SmMYB52 activated the transcription of *SmTAT1*, *Sm4CL9*, *SmC4H1*, and *SmHPPR1* (Figure 6D). Strong firefly luciferase signals were observed when the effector vector and recombinant reporter vector were cotransformed into tobacco leaves (Figure 6E). These findings provided supporting evidence that SmMYB52 binds directly to and activates the promoters of *SmTAT1*, *Sm4CL9*, *SmC4H1*, and *SmHPPR1* to upregulate the biosynthesis of phenolic acids.

## 3. Discussion

In the present study, we characterized the function of *SmMYB52* in *S. miltiorrhiza* by generating transgenic plantlets with an enhanced or reduced expression of *SmMYB52*. *SmMYB52*-overexpression lines showed obvious phenotype changes with inhibited root growth and significantly increased SalB and RA levels, while *SmMYB52*-knock down lines exhibited the opposite phenotypes. We concluded that *SmMYB52* was a strong regulator for promoting the accumulation of SalB and simultaneously inhibiting root growth.

Our previous study indicated that *SmMYB52* tended to cluster with *AtMYB93* [26]. The root phenotype in *SmMYB52*-silencing or *SmMYB52*-overexpressing lines was consistent with that in *Atmyb93* mutants or *AtMYB93*-overexpression lines [12]. *AtMYB93* was induced by auxin [12], while *SmMYB52* was unexpectedly suppressed by the exogenous IAA treatment (Figure 2A). The opposite expression responses of *SmMYB52* and *AtMYB93* to IAA were possibly due to the application of different concentrations of exogenous IAA. In the present study, 100 μM IAA was used to spray the seedlings of *S. miltiorrhiza*, while the Arabidopsis seedlings were treated with 1 μM or 10 μM IAA. Whether *SmMYB52* is induced by low concentrations of IAA should be further investigated.

Auxin plays important roles in the control of root development and structure [31]. There is a dose-dependent effect of IAA on root growth, where very low or very high concentrations have an inhibitory effect on root growth [32]. The concentrations of endogenous auxin vary at different stages of root development, where higher concentrations are required for root induction, including the formation of adventitious roots [33,34]

In this study, the overexpression of *SmMYB52* inhibited root growth, which was likely due to the suppression of the biosynthesis of IAA. The IAA biosynthetic pathway (trp-dependent pathway) involves at least five biosynthetic enzyme genes including *TAA1*, *TIR2*, *TAR1*, *AIM1*, *YUC1*, and *AAO1* [35]. We found that overexpressing *SmMYB52* in *S. miltiorrhiza* significantly decreased the concentration of endogenous auxin by suppressing the transcripts of *SmTAA2*, *SmAIM1*, *SmAAO1*, and *SmYUC1* (Figure 2B,D). The overexpression of *OsYUCCA1* promoted the accumulation of IAA and the formation of adventitious roots in rice. In contrast, a knockdown of the *OsYUCCA1* inhibited root formation and elongation [27]. Similarly, the significantly increased transcripts of *SmYUC1* in *SmMYB52*-silencing lines possibly promoted the IAA level and root growth. Thus, we proposed that SmMYB52 inhibited root growth by suppressing the biosynthesis of IAA in *S. miltiorrhiza*.

JA is an important hormone that regulates plant growth, the responses to environmental stresses, and is directly involved in root growth [36]. When treated with exogenous MeJA root, growth was inhibited in *A. thaliana* seedlings. Further, MeJA treatment promoted the production of saikosaponin and inhibited adventitious root development in *Bupleurum kaoi* [37]. The effects of the exogenous application of MeJA on root growth and active compounds were reported for *S. miltiorrhiza* [38].

In this study, the overexpression of *SmMYB52* inhibited root growth and increased the SalB level, which was similar to the phenotype induced by the exogenous MeJA treatment. We speculated as to whether the endogenous JA level was altered in the roots of the transgenic lines of *S. miltiorrhiza*. Our results revealed that the JA level was increased in *SmMYB52*-overexpressing lines, while it was decreased in the *SmMYB52*-silencing lines (Figure 3B). Thus, the increased level of JA was another factor involved in the reduction of root growth in *SmMYB52*-overexpressing *S. miltiorrhiza*. JA biosynthesis involves at least five biosynthetic enzymes genes including *JAR1*, *LOX*, *AOC*, *AOS*, and *OPR3* [39]. Our qRT-PCR results indicated that the overexpression of *SmMYB52* significantly increased the level of endogenous JA by promoting the transcripts of *SmLOX3*, *SmAOC*, *SmOPR3*, and *SmJAR1* (Figure 3D).

Numerous studies demonstrated that JA-auxin crosstalk is critical for root growth and development [40] Auxin regulates the initiation of adventitious roots by modulating JA homeostasis in Arabidopsis [41], and the JA-mediated inhibition of root development is auxin-dependent [42]. The auxin-inducible genes *GH3.3*, *GH3.5*, and *GH3.6* might inactivate an inhibitor of adventitious rooting, and the JA level of the *gh3.3-1gh3.5-2gh3.6-1* mutant, in which the number of adventitious roots was reduced, was twice that of the wild type [41].

In the present study, SmMYB52 positively regulated the biosynthesis of JA, while it negatively regulated IAA biosynthesis (Figues 2 and 3). We speculated that SmMYB52 may inhibit root growth via synergies between the JA and auxin signaling pathways in *S. miltiorrhiza*. We found that the MYB-binding sites existed in the promoters of both IAA and JA biosynthesis genes (Figure S2). Whether SmMYB52 directly activates or suppresses their expression by binding to their promoters should be investigated in the future.

In *S. miltiorrhiza*, some of the R2R3-MYB TFs, including *Sm*MYB1 [19], *Sm*MYB36 [23], *Sm*MYB39 [43], *Sm*MYB97 [21], *Sm*MYB98 [44], and *Sm*MYB111 [22], were reported to regulate the biosynthesis of SalB either positively or negatively. R2R3-MYB TFs typically regulate target genes by directly binding to the elements of the promoters [21]. As key enzyme-encoding genes involved in the SalB biosynthesis pathway, *SmTAT1* and *SmPAL1* might be directly activated by *Sm*MYB97 [21], whereas *SmCYP98A14* could be directly activated by *Sm*MYB1 [19].

Here, we verified that *Sm*MYB52 was a positive regulator for the biosynthesis of RA and SalB (Figure 4C,D). Y1H and dual-luciferase assays showed that *Sm*MYB52 promoted the biosynthesis of SalB by directly binding to and activating the promoters of *SmTAT1*, *SmHPPR1*, *Sm4CL9*, and *SmC4H1* (Figure 6). *Sm*MYB52 (a MeJA-responsive TF) not only directly regulates the biosynthesis of SalB, but is also involved in the accumulation of JA, which is recognized as stress hormone and participates in the regulation of secondary metabolites. To our knowledge, this is the first report that focuses not only on the accumulation of active compounds, but also on root growth in *S. miltiorrhiza*. *Sm*MYB52 plays diverse functions in *S. miltiorrhiza*, which is a potential regulator of growth-defense trade-offs and auxin-JA crosstalk during root development.

**Figure 7:**
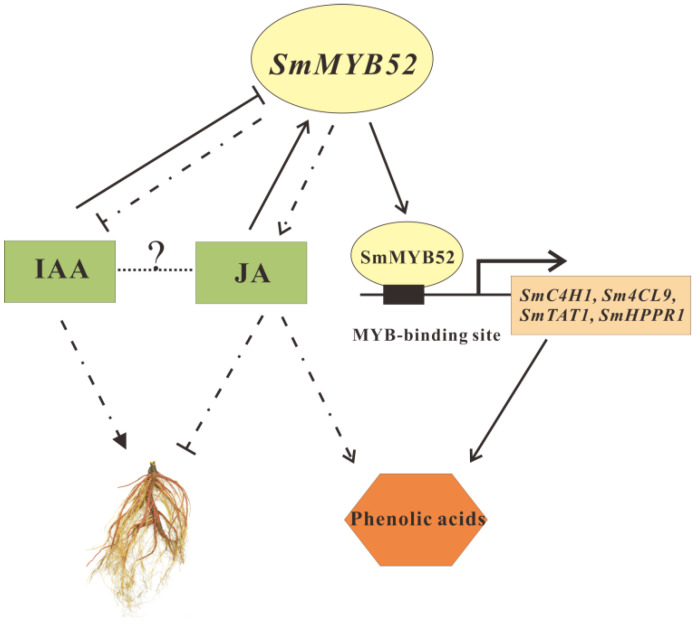
Working model of *SmMYB52* regulating root growth and biosynthesis of phenolic acids. Overexpression of *SmMYB52* suppresses biosynthesis of IAA and promotes biosynthesis of JA, which results in inhibition of root growth. *SmMYB52* activates its target genes (e.g., *SmC4H1* and *Sm4CL9*) to further modulate accumulation of phenolic acids. Arrows represent positive regulation; blunt ends represent negative regulation. Dotted line indicates putative pathway.

Derived from our results and previous reports, we proposed a working model that *Sm*MYB52 regulates root growth and the biosynthesis of phenolic acids (Figure 7). In this model, MeJA- and auxin-responsive *SmMYB52* inhibits root growth by downregulating and upregulating the biosynthesis of IAA and JA, respectively. *Sm*MYB52 regulates the concentration of SalB in two ways: (1) *Sm*MYB52 directly binds to and activates the promoters of some key enzyme-encoding genes, and then positively regulates the biosynthesis of phenolic acids. (2) *Sm*MYB52 participates in SalB biosynthesis via JA-mediated regulation. Further significant research is required to perfect this model, which should include how *Sm*MYB52 regulates the biosynthesis of IAA and JA.

## 4. Materials and Methods

### 4.1. Experimental Materials, Growth Conditions, and Phytohormone Treatments

*S. miltiorrhiza* seeds were sterilized with Hgcl_2_ and cultured on Murashige and Skoog basal medium (Solarbio, Beijing, China) for the transformation experiments [45]. The identified transgenic lines were continued to cultivate in the 10 cm diameter sterile culture bottles on 400 mL MS medium. *Nicotiana benthamiana* seedlings were cultured in a growth chamber at 25 ± 2 °C under a 16 h light: 8 h dark photoperiod for 5 weeks prior to a transient expression assay [46].

The leaves of two-month-old *S. miltiorrhiza* plantlets were treated with MeJA and IAA (Sigma-Aldrich Corp., St. Louis, MO, USA), respectively. According to the previous studies [21,47], the stock solutions of MeJA and IAA were diluted to a final concentration of 100 μM, which were then sprayed at a volume of ~1.5 mL on the leaves of each plantlet. The whole plantlets were collected at the time points of 0, 30 min, 1, 2, 3, 9, and 12 h following phytohormone treatment, after which the total RNA was extracted and converted to 1st-strand cDNA for qRT-PCR (Vazyme, Nanjing, China), and housekeeping gene *SmUbiquitin* was used as an internal reference. The treatments were performed in three independent biological replicates.

All chemical reagents used in the experiment were obtained from Sigma Chemical Co. (St. Louis, MO, USA). The standard RA and SalB used in the HPLC were purchased from the National Institute for the Control of Pharmaceutical and Biological Products (Beijing, China). All primers are shown in Table S1.

### 4.2. Construction of Plant Expression Vectors and Plant Transformation

The full-length open reading frame (ORF) of *SmMYB52* was amplified using the 207-SmMYB52-F/R primers containing the *attB1/attB2* sites, which were then recombined into the donor vector pDONR207 via the standard BP reaction to generate the entry vector pDONR207-*SmMYB52*, according to the Gateway manufacturer’s protocol (Invitrogen, Carlsbad, CA, USA). Subsequently, *SmMYB52* was further recombined into the pEarleyGate 202 via LR reaction to obtain the pEarleyGate 202-*SmMYB52* overexpression vector. To silence the *SmMYB52* in *S. miltiorrhiza*, an RNAi vector pSmMYB52-RNAi was synthesized as described previously [22]. Briefly, we designed a pair of amiRNA primers, RNAi-SmMYB52-F/R using P-SAMS software (http://p-sams.carringtonlab.org/) (accessed on 1 August 2018). Then the primers were denatured, cooled, and annealed, and the formed amiRNA cassette was then recombined into the pMDC123SB-AtMIR390a-B/c vector via *BsaI* and T4 DNA ligase (TaKaRa, Beijing, China). Through *Agrobacterium*-mediated transformation, *SmMYB52*-overexpressing and -silencing transgenic plants were acquired as previously described [45]. In parallel, regenerated plantlets without *Agrobacterium*-mediated transformation were used as a control.

### 4.3. Selection of Transgenic Plantlets

To identify the positive transgenic lines, the genomic DNA was isolated from the transgenic lines and used as templates to amplify the CaMV35S promoter sequence using primer pairs 35S-F/R and 2 × 35S-F/R for *SmMYB52*-overexpressing and *SmMYB52*-silencing lines, respectively. The total RNA was extracted from the roots of two-month-old transgenic and control lines and converted to 1st-strand cDNA. The expression levels of *SmMYB52* and the enzyme-encoding genes involved in IAA, JA, and SalB biosynthetic pathways were detected by qRT-PCR, and their relative expression levels were calculated using the 2^−ΔΔCt^ method [48]. After selection, the transgenic lines of the T1 generation were collected as materials for the following assays.

### 4.4. Determination of IAA and JA Concentrations

The endogenous hormone levels of IAA and JA were calculated using the Plant IAA/JA ELISA Kit (mlbio, Shanghai, China), following the manufacturer’s protocols. Briefly, 0.1 g fresh roots from each of two-month-old transgenic line and the control line were weighed, to which 900 μL of a PBS buffer was added after being fully ground. The homogenates were then centrifuged for 10 min at 6000 rpm, and the supernatants were transferred to new tubes. To standards and samples was added 100 μL enzyme conjugate, and then incubated for 60 min at 37 °C. Subsequently, the substrate was added to the standards and samples and then incubated in the dark for 15 min, after which a stop solution was added to each well. Finally, the optical density (OD) was measured at 450 nm. According to the concentration of the calibration standards and the corresponding absorbance, a standard curve was obtained, and the hormone content of the samples was calculated.

### 4.5. Detection of Secondary Metabolites

The roots of two-month-old transgenic and control lines were dried to constant weight at 25 ± 2 °C and used for detection. The total levels flavonoid and phenolic were determined via methods as previously described [49].

To extract SalB and RA, a 500 μL solution (methanol: acetone, 7:3, *v*/*v*) was added to 100 mg dry root powder for ultrasonic extraction at 30 °C and 100 Hz for 1 h, followed by centrifugation at 6000 rpm for 3 min (Eppendorf, Hamburg, Germany). Afterwards, the supernatant was transferred into a new 1.5 mL centrifuge tube and the extraction was repeated, while the supernatants were combined twice for the determination of phenolic acids. The SalB and RA concentrations were measured via HPLC using an Agilent ZORBAX SB-C18 column (250 × 4.6 mm, 5 μm) with an UltiMate 3000 HPLC System coupled with DAD detector (Thermo Fisher Scientific, Waltham, MA, USA). We used standards of RA, Sal B in HPLC experiments, and the detection wavelength was 280 nm. All separations were carried out at a constant temperature of 30 °C, with the specific process and parameter settings being performed as described previously [22]. The metabolite contents were calculated based on the retention time and peak area of the standards (in the chromatograms).

### 4.6. Y1H Assays

The full-length *SmMYB52* ORF was amplified by PCR using Y1H-SmMYB52-F/R primers that contained *Eco*RI and *Bam*HI restriction sites, which were then fused to pGADT7-Rec2 with T4 DNA ligase (TaKaRa, Beijing, China). The 1449-bp and 1652-bp promoter regions of *SmHPPR1* and *SmC4H1*, respectively, were amplified and inserted into the pHIS2. The pHIS2–*SmTAT1* and pHIS2–*Sm4CL9* were constructed in our earlier study [29,50].

According to a previous method [51], these recombinant plasmids were transferred to yeast strain Y187 (Weidi Biotechnology, Shanghai, China), followed by cultivation on SD/-Leu/-Trp medium (DDO) (Solarbio, Beijing, China) for 3 days at 29 °C, and then screened on SD/-Leu/-Trp/-His medium (TDO) (Solarbio, Beijing, China), supplemented with 60 mM 3-amino-1,2,4-triazole (3-AT) (Solarbio, Beijing, China) to determine whether the SmMYB52 interacted with the promoters of key enzyme-encoding genes [21]. The pGADT7-p53+ p53-HIS2 and p53-HIS2+pGADT7 empty vector were served as positive control and negative control, respectively.

### 4.7. Dual-luciferase Assay

The *SmMYB52* was amplified by PCR using 62sk-SmMYB52-F/R primers and inserted into the pGreenII62SK vector, which served as the effector. The promoter regions of *SmHPPR1* and *SmC4H1* were then inserted into the pGreenII 0800 empty vector. The recombinant vectors of *pSmTAT1*-pGreenII 0800 LUC and *pSm4CL9*-pGreenII 0800 LUC were constructed in our previous reports [29,50].

The constructed effector and reporter vectors were then cotransferred into tobacco leaves via *Agrobacterium*-mediated transformation [29]. Three days following infiltration, the activities of LUC (firefly luciferase) and renilla luciferase (REN) were determined via a dual-luciferase reporter gene assay kit (Beyotime, Shanghai, China), then the promoter activity was calculated according to the the ratio of LUC to REN. In addition, the LUC activity was imaged using IVIS Spectrum (Xenogen, Alameda, CA, USA) via substrate beetle luciferin (Promega, Madison, WI, USA) applied on the leaves of tobacco. The SmMYB52+pGreenII 0800-LUC empty vector served as the negative control, while SmbHLH37+*pSmTAT1* served as the positive control [29].

## 5. Conclusions

In this study, we verified that *SmMYB52* was a positive regulator for the biosynthesis of RA and SalB. SmMYB52 promoted the biosynthesis of SalB by directly binding to and activating the promoters of *SmTAT1, SmHPPR1, Sm4CL9,* and *SmC4H1*. Meanwhile, according to the phenotypes and the level of endogenous IAA and JA, we found that root growth was inhibited with higher *SmMYB52* transcript levels, which were possibly due to the effects of SmMYB52 on the biosynthesis of indole-3-acetic acid (IAA) and jasmonate acid (JA).

## Data Availability

No new data were created or analyzed in this study. Data sharing is not applicable to this article.

## Supplementary Materials

The following are available online at https://www.mdpi.com/article/10.3390/ijms22179538/s1.
