# Supplementary material for: R2R3-MYB Transcription Factor SmMYB52 Positively Regulates Biosynthesis of Salvianolic Acid B and Inhibits Root Growth in Salvia miltiorrhiza
Source: Int J Mol Sci. 2021 Sep 2;22(17):9538. doi: 10.3390/ijms22179538 (PMC8431584; doi:10.3390/ijms22179538)
Supplement: Supplementary file 1 [file ijms-22-09538-s001.zip › ijms-1325131-supplementary.pdf]

Table S1 Primers used in experimental procedures.

Gene ID*	Primers	Sequence(5'-3')	Tm(℃)
**Primers used for qRT-PCR**			
KF059406	RT-MYB52-F	CTCCGATTTCTGATGATAGTGGTGT	60.5℃
	RT-MYB52-R	ATGTTTTTGGATGTATTTGGTGAGG	
SMil_00024925-RA_Salv	RT-TAT1-F	CAACTGCTGGTCTTCCACAAAC	59.7℃
	RT-TAT1-R	GCGAGCCAAAACGGACA	
SMil_00002851-RA_Salv	RT-TAT2-F	CGGAGATCCATCCGCCTTCC	62℃
	RT-TAT2-R	CGGGAATACCGACGGTGGAG	
SMil_00020694-RA_Salv	RT-TAT3-F	TGCTGAAACTGCCAAGAGGCT	61℃
	RT-TAT3-R	CCGGGCACCAACCATCTCTT	
SMil_00012897-RA_Salv	RT-PAL3-F	CTCCACCCGTCGAGGTTCTG	62℃
	RT-PAL3-R	TCTGCATGAGCGGGTACGTG	
SMil_00002680-RA_Salv	RT-HPPR1-F	TGACTCCAGAAACAACCCACATT	61℃
	RT-HPPR1-R	CCCAGACGACCCTCCACAAG	
SMil_00013707-RA_Salv	RT-HPPR2-F	CTTACAGCGTCGGCCTCGAT	61℃
	RT-HPPR2-R	GTGGTCAGAGCCAGCCCAAT	
SMil_00013867-RA_Salv	RT-HPPR3-F	GCGCTACCGCTCCTTGAGAT	60.9℃
	RT-HPPR3-R	CACAAATCCGCCGCGAAGTC	
SMil_00000716-RA_Salv	RT-C4H1-F	CCAGGAGTCCAAATAACAGAGCCG	62℃
	RT-C4H1-R	GCCACCAAGCGTTCACCAAGAT	
SMil_00008129-RA_Salv	RT-4CL1-F	TCACCCATGCCGGATTCGAG	62℃
	RT-4CL1-R	AGATCGCGCCGATGAAGGAG	
SMil_00018588-RA_Salv	RT-4CL9-F	GAGCACAGAAGAAGAGTATGATGGG	61℃
	RT-4CL9-R	AGCAGCGAGCCGATCTCAAA	
SMil_00025190-RA_Salv	RT-RAS1-F	CCAAAGTCAATTATGCCAAGGG	60.1℃
	RT-RAS1-R	GTCGGATAGGTGGTGCTCGT	
	RT-CYP98A14-F	ACGTGCGTGTTGCTACGAGAC	61℃
	RT-CYP98A14-R	CGTCGCCAGTGCTGCAACTAA	
SMil_00027821-RA_Salv	RT-LOX3-F	TTCTTCTGTTTCACTGCCTCCT	59℃
	RT-LOX3-R	TAACATACCGTGTACCTAACTAC	
SMil_00002529-RA_Salv	RT-AOS-F	CCCACGCATGAAAGTACACCAGA	61.3℃

	RT-AOS-R	ATCCTTCCCTGAACCCTCACTCC	
KF220568.1	RT-OPPR3-F	TTGAAGCAGGATTTGATGGCA	61.5℃
	RT-OPPR3-R	GGCGAGTTTGGAACCACAGG	
SMil_00003673-RA_Salv	RT-JAR1-F	GATAACTCATCCGTCATCACCG	59.7℃
	RT-JAR1-R	AACGAGGTCTTGTAAATTTGCATG	
SMil_00008622-RA_Salv	RT-TAA1-F	GCCTCAATACACCGCCATC	59℃
	RT-TAA1-R	CGTCTCGTACAATCGCCCA	
SMil_00008177-RA_Salv	RT-TAA2-F	CTACTCCTGCTATCCACAGATAGCG	61℃
	RT-TAA2-R	CCAACTCTATGTAAGCCTCATCTTT	
SMil_00009945-RA_Salv	RT-AIM1-F	CTCCTTCCGTTATCTCAGCCT	59℃
	RT-AIM1-R	GTACCCTCTTCCAATCAGCACTTC	
SMil_00002467-RA_Salv	RT-YUC1-F	CGACTCTGTTATCTTGGCTACGGG	61℃
	RT-YUC1-R	GAACCCAACTGCGTAAATCCCA	
SMil_00014441-RA_Salv	RT-AAO1-F	GTCTGTGCTGTCAATGGAGCCCTTC	62.3℃
	RT-AAO1-R	CTTTTGCGCCACATGTTAGAACGAT	
	RT-Ubiquitin-F	ACCCTCACGGGGAAGACCATC	61℃
	RT-Ubiquitin-R	ACCACGGAGACGGAGGACAAG	
	**Primers used for vector construction**		
KF059406	SmMYB52-F	ATGGGAAGATCTCCGATTTC	59℃
	SmMYB52-R	ATTATGTACTATGGAATGTCATGGA	
	207-SmMYB52-F	GGGGACAAGTTTGTACAAAAAAGCAGGCTTCATGGGAAG ATCTCCGATTTCT	60℃
	207-SmMYB52-R	GGGGACCACTTTGTACAAGAAAGCTGGGTCTGGAATGTC ATGGAAAATAGGAT	
	Y1H- SmMYB52-F	GGAATTCATGGGAAGATCTCCGATTTC	59.6℃
	Y1H- SmMYB52-R	CGGGATCCCCTATGGAATGTCATGGAAAATAGGAT	
SMil_00000716-RA_Salv	Y1H-SmC4H1-F	CCGGAATTCGTTGATATGTCAAGTTTGAGACAAG	57.6℃
	Y1H-SmC4H1-R	CGAGCTCATGGTTTGCGGTCTGCAGT	
SMil_00002680-RA_Salv	Y1H-SmHPPR1-F	TCCCCCGGGGGAAACATCATCTGCAGTGCCTA	58.5℃
	Y1H-SmHPPR1-R	CGACGCGTAGATGGTGTTTGAGTGGTGGTTG	
	62sk-SmMYB52-F	GGAATTCATGGGAAGATCTCCGATTTC	59℃
	62sk-SmMYB52-R	CGGGATCCCCTATGGAATGTCATGGAAAATAGGAT	
	LUC-SmC4H1-F	CCGCTCGAGGTTGATATGTCAAGTTTGAGACAAG	57.6℃
	LUC-SmC4H1-R	CGCGGATCCATGGTTTGCGGTCTGCAGT	

LUC-SmHPPR1-F	CCGCTCGAGGGAAACATCATCTGCAGTGCCTA	58.5℃
LUC-SmHPPR1-R	CGCGGATCCAGATGGTGTTTGAGTGGTGGTTG	
35S-F	AAAGGCCCAGCAGTGATC	58℃
35S-R	GAAGGATAGTGGGATTGTGC	
RNAi-SmMYB52-F	TGTATCCAGGGGCCTTTCTTCACAGATGATGATCACATTC<br>GTTATCTATTTTTTCTGTGAAGAACGGCCCCTGGA	65℃
RNAi-SmMYB52-R	AATGTCCAGGGGCCGTTCTTCACAGAAAAAATAGATAAC<br>GAATGTGATCATCATC TGTGAAGAAAGGCCCCTGGA	
attb1	ACAAGTTTGTACAAAAAAGCAGGC	58.5℃
attb2	ACCACTTTGTACAAGAAAGCTGGGT	
2×35S-F	GGTCAACATGGTGGTGC	58℃
2×35S-R	AGGGTCTTGCGAAGGATAG	

* These genes were retrieved from the web portal at http://www.ndctcm.org/shujukujieshao/2015-04-23/27.html.

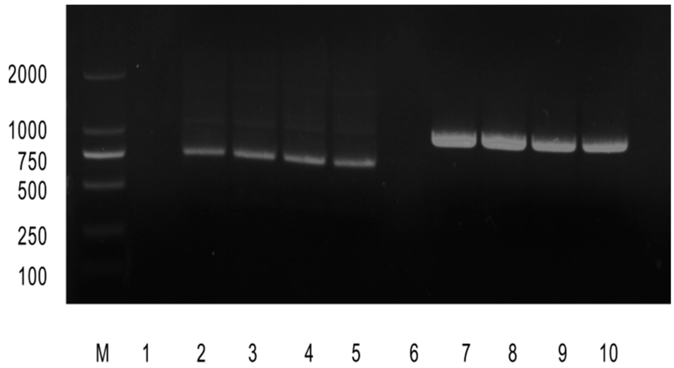

Figure S1. Identification of *SmMYB52*-expression and *SmMYB52*-sliencing transgenic lines by PCR.

Lanes: M, DL2000 DNA marker; 1, wild-type plants as negative control; 2 and 7, pMDC123SB-AtMIR390a-B/c empty vector and pEarleyGate 202 empty vector; 3-5 , *SmMYB52*-sliencing transgenic lines; 8-10, positive *SmMYB52*-expression transgenic lines;. Overexpressing lines comprised a 926-bp fragment of the CaMV 35S promoter and RNAi lines contained a 720-bp fragment as expected.

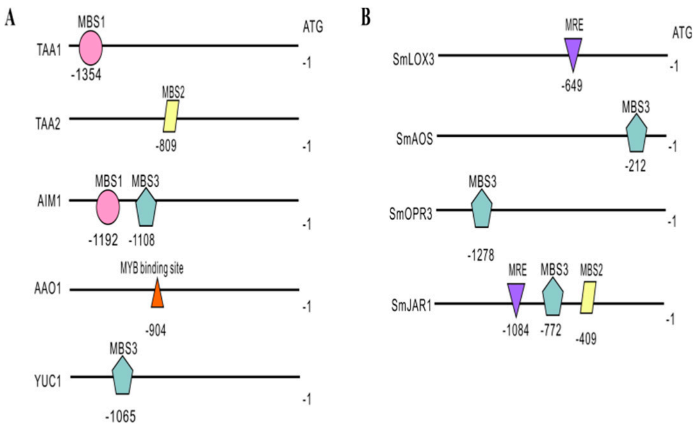

Figure S2. Distribution of the MYB-binding sites in the promoter regions of the IAA (A) and JA (B) biosynthesis enzyme-encoding genes.

```
               CTTTCTCACGAAAAATATATTTCTTGCTATAACCACACATTCATGGTTAATAGTGTTTTGTGTCCCCAACTTTCA
               GCATCTTTCATAAAATGTCCTCAACTTTCATGTTCTCTTCAAAACCCTTCAATTTTCAGCTTTTTCCATAAAATG
TGACG-motif    TATGACGATCCAAATTCTGGTGATGAAAAGATAATTCATCGCACCAGAGTCTTAGGTGAGTCTTTTTTTCCAA
               CTCGCCATATGAAACAACGTCTTTTTTATGCTAAGTGAAAAAAAAATGAAAATTTAACAATCTCTCCATCTCTCA
               TCATCGTTAATCACCGCAAAAAACTCTCCTTCCAAAATAAGTCGATCTCTTATCCAAAATCGGTGAAAGTCGAT
               GTTCTACCACTGCTTCTTCTGTTGTTAGCCTTGCTCCATCATCACCAAAGTGAAATTAATAGCATCAAAAATTG
               GTGGGTTGAATCTCACAATGCAAATTTAGAAGTAAGATTCATATGGCAACTGTGGTAAGTGGCGGTCTCTTG
               GTCGGAGTTAATTAGAAGGAAGATGGTTGCAGATTTTGAAGGTGATGTAGATGAGATTTTGTAGAAAGAGA
TGACG-motif    TGAATGACGTAGTTTTAATGTTTAATTAGGTGGTAATTTTTTTAAAAGTTAAATTTTACGTGAGTCTTGATGAA
               ATGAATCGTCTTTTCATTACTAAATTTTACTATATAGCGAAACATTTTATAAACAATACTGAAAATAAAGAATTT
               TTAAGAAATTAAGAATGAAATTAGAAACATTGTATGAAAAATATTCATGTCGGGACATACTTCACCCATATATTT
               ACATAAAACCTGACGTGAATTTATGAAAAAAGAAATCACCATGCAAAATATGCAATCAGTGACGACGATTGC
CGTCA-motif    GTGCATGGAGTGTGTTGTTCTGACCAGATGGTAGCCGCCACCTGTTCCTCATATTGACTTCTTCCTTTCAACC
               TTTTCTACTCTATTTTCTTATCATTGTTGATTTTGAAACAACACTAACTCCCAAATTTGCCCCTATTCCTTTTTTG
               GTTTACTTTGTCTTAATTTCCAAAAAAAATTGTTCTTTTTCCTCCATGGTTTTTGTTGGCTTCTTTTTTAGTATTAT
               ATAAAATTTAGGGACAAGATACAAAAACGTCCTCCACATTTGTATTGATACGCAAATATTCTCTAGTGTATTTT
               GGTGCAAAAAAAGCTAATTAAACCTCAAATAAATATTGCAGTCACAGCTGTTTAATTTAAATCGATTTTTCCCC
               TATAATTAATTAATATATAAAAAGGGTAGTGATAATTGATGAAGTGGCAGAATCATGTGGGCCTCCCTCCTACA
               GAAGCTATGTCCCATATCATAGGACATGCTAGAAGAAGACAAACGAGGGGAAACAATAGTTGCACTAATATAT
               ATATATATATATAGTAGTTATTTGGTAGGATTAATTGTAAGAAGCAATCGAAAGCAGAATCAAGTATAGTTTAG
               AGAGAGAAAGGAGAGGGCCGAG
```

Figure S3. Distribution of the MeJA-responsiveness elements in the promoter region of *SmMYB52*.
